# Daily Intake and Serum Levels of Copper, Selenium and Zinc According to Glucose Metabolism: Cross-Sectional and Comparative Study

**DOI:** 10.3390/nu13114044

**Published:** 2021-11-12

**Authors:** Vishwanath Pattan, Maria Mercedes Chang Villacreses, Rudruidee Karnchanasorn, Ken C. Chiu, Raynald Samoa

**Affiliations:** 1 Department of Medicine, Division of Endocrinology, Diabetes and Metabolism, SUNY Upstate Medical University, Syracuse, NY 13210, USA; pattanv@upstate.edu; 2 Department of Clinical Diabetes, Endocrinology, and Metabolism, Diabetes and Metabolism Research Institute, City of Hope National Medical Center, Duarte, CA 91010, USA; mchangvillacreses@coh.org (M.M.C.V.); rsamoa@coh.org (R.S.); 3 Division of Endocrinology, Department of Medicine, University of Kansas Medical Center, Kansas City, KS 66160, USA; rudruidee@gmail.com; 4 Division of Endocrinology, Metabolism and Nutrition, Department of Internal Medicine, Harbor-UCLA Medical Center, Torrance, CA 90509, USA

**Keywords:** trace element, copper, selenium, zinc, diabetes mellitus, glucose metabolism

## Abstract

Trace elements play an important role in metabolism. We compared the daily intake and serum concentrations of copper (Cu), selenium (Se), and zinc (Zn) across a spectrum of glucose tolerance status in a representative U.S. population. Daily intake and serum concentrations of Cu, Zn and Se in 5087 adults from the 2011–2016 National Health and Nutrition Examination Survey (NHANES) were examined and compared to normal (NGT) and abnormal (AGT) glucose tolerance and the presence of diabetes mellitus (DM). Other than Zn deficiency (21.15%), the prevalence of Zn, Se, and Cu excess and Se and Cu deficiency were low (<4.00%). As compared to the NGT group, Cu and Se supplementation was higher in the AGT and DM groups (*p* < 0.0001 for all). Serum Se and Zn, but not Cu, concentrations were highly correlated with daily intake (*p* < 0.0001 for both). As compared to the NGT group, serum Cu concentration was highest in the AGT group (*p* = 0.03), serum Se concentration was highest in the DM group (*p* < 0.0001), and serum Zn concentration was highest in the AGT group (*p* < 0.0001). Serum Se and Zn concentration was correlated with daily Se and Zn intake. Even within the reference range for serum Cu, Se, and Zn concentrations, a higher serum concentration of Cu, Se, and Zn was associated with abnormal glucose metabolism. Although the casual relationship remains to be elucidated, these data suggest caution in Cu, Se and Zn supplementation in non-deficient individuals.

## 1. Introduction

Trace elements are essential for proper growth, development, and physiology [1]. Experts recommend daily intake of these minerals in small quantities between 1 to 100 mg [2]. Foods grown in low content soil can result in trace element deficiencies in some individuals, with clinical consequences. For example, Keshan disease manifests in children and women of child-bearing age as cardiac myopathy, primarily in rural China where the soil is deficient in selenium [3]. It is necessary to maintain a proper balance of trace elements as they are salient components in many biological pathways. However, excessive amounts are implicated in pathological processes, such as hemochromatosis from iron overload [4].

Endocrine pathways may be significantly impacted by the availability of trace elements. Alteration in levels of trace elements was noted in individuals with type 2 diabetes mellitus [5]. Trace element deficiency was reported to promote insulin resistance [6] and beta cell dysfunction [5,7]. More specifically, copper (Cu) [8], selenium (Se) [9], and zinc (Zn) [7] were implicated in the pathogenesis of diabetes and glucose dysregulation. Based on these studies, widespread trace element supplementation was proposed to decrease the risk of developing diabetes. However, a paucity of data exists regarding the possible relationship between trace element levels and glucose tolerance. The controversy is mainly from the small sample size and lack of or inadequate assessment of daily intake, as noted from the reported association studies [10,11,12,13,14]. Thus, additional studies with a better study design and a larger sample size are needed to address these issues properly.

The National Health and Nutrition Examination Survey (NHANES) is a national survey program designed to assess the nutritional and health status of a highly representative population in the United States since the 1960s, which consists of a survey of comprehensive dietary intake nationwide. To avoid problems related to sample size and lack of adequate assessment of dietary nutrient intake, we compared daily intake and serum concentrations of Cu, Se, and Zn on a spectrum of glucose tolerance status in a highly representative U.S. population.

## 2. Materials and Methods

### 2.1. Study Subjects

This is a cross-sectional study to compare daily intake and serum concentrations of Cu, Se, and Zn in three states of glucose tolerance, namely normal glucose tolerance (NGT), abnormal glucose tolerance (AGT), and diabetes mellitus (DM) as defined in Section 2.2. The study analyzed de-identified data from subjects enrolled in the National Health and Nutrition Examination Survey (NHANES) from 2011 through 2016. The study has been conducted in accordance with the Declaration of Helsinki. The study was approved by the Research Ethics Review Board of the National Center for Health Statistics. Informed consent was obtained from each participant at entry to the survey. NHANES uses a very complicated sampling scheme and assigns sample weight individuals for analysis. For each subsample study, subjects were carefully selected using a similar complicated sampling scheme for representative samples with subsample specific gravity for analysis. Thus, the weighted analyses, recommended by NHANES and performed in this study, provided a representative result that can be generalized to the US population. Due to the NHANES study design, no information was collected to define the types of diabetes. Since type 1 diabetes alone accounts for no more than 8% of diabetes in the United States, the majority of diabetic patients had type 2 diabetes. Due to the complex sampling scheme of the NHANES study design, no other type of chronic disease was excluded in the design of this study. Only those data with dietary recall status defined as reliable and met the minimum criteria by the NHANES were included in this study. The methods were developed and validated by the NHANES since 2005. The NHANES did not provide the reference ranges for daily intake for Cu, Se, and Zn.

**Figure 1:**
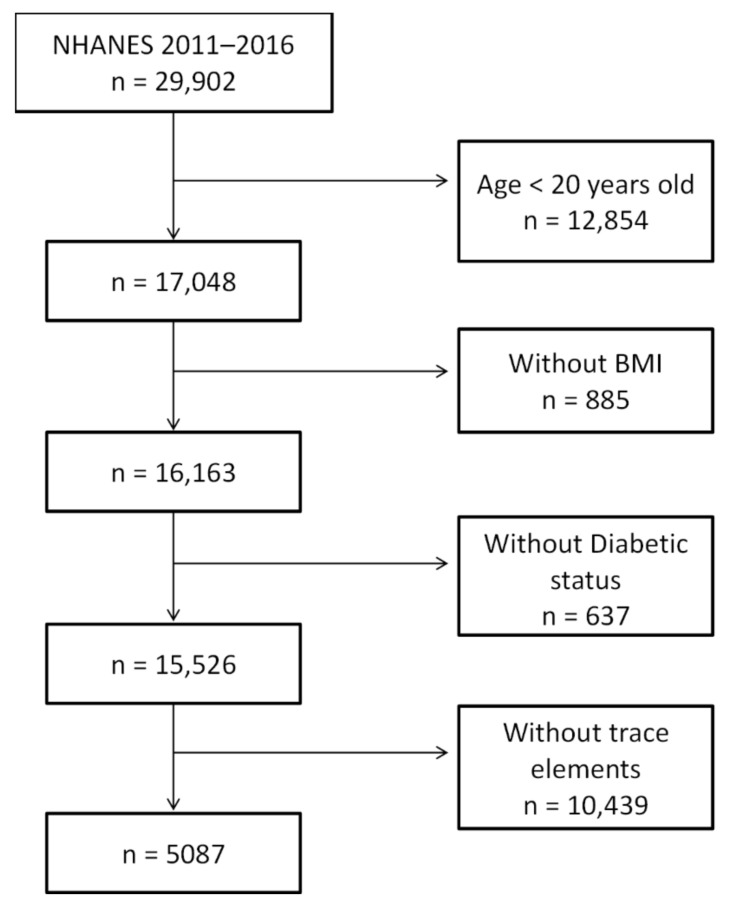
Sampling scheme.

Specifically, we only included (1) adult subjects (≥20 years), since some key questionnaires were not administered for subjects less than 20; (2) subjects with BMI data available, since BMI has a major impact on glucose metabolism; (3) defined states of glucose tolerance by medical history, medication uses, hemoglobin A1c, fasting and 2-h postchallenged glucose concentration; (4) subjects with dietary data available; and (5) subjects with available measurements of serum Cu, Se, and Zn concentrations. We excluded (1) subjects who were less than 20 years of age; and (2) subjects who were missing any of above critical information. The sampling scheme is shown in Figure 1, with 5087 participants. The subjects were divided into three states of glucose tolerance, based on the definition outlined in Section 2.2. Definition of glucose tolerance states", for the investigation of daily intake and serum Cu, Se, and Zn concentrations. The clinical characteristics of the study subjects are summarized in Table 1.

### 2.2. Definition of Glucose Tolerance States

States of glucose tolerance were defined based on the current definition from the American Diabetes Association [15]. DM was defined by one of the following criteria: (1) told that they have diabetes by a health care provider; (2) are taking any anti-diabetic medication; (3) HbA1c ≥ 6.5% (48 mmol/mol); (4) fasting plasma glucose (FPG) concentration ≥ 126 mg/dL (7.0 mmol/L); or (5) 2-h post-challenged plasma glucose concentration ≥ 200 mg/dL (11.1 mmol/L). After exclusion of diabetic subjects by the above criteria, AGT was defined by one the following criteria: (1) HbA1c ≥ 5.7% (39 mmol/mol); (2) FPG ≥ 100 mg/dL (5.6 mmol/L); or (3) 2-h post-challenged plasma glucose concentration ≥ 140 mg/dL (7.8 mmol/L). After exclusion of diabetic and AGT subjects, NGT was defined by the presence of (1) HbA1c < 5.7% (39 mmol/mol); (2) FPG < 100 mg/dL (5.6 mmol/L); and/or (3) 2-h post-challenged plasma glucose concentration < 140 mg/dL (7.8 mmol/L).

### 2.3. Laboratory Methods

Plasma glucose concentration: Plasma glucose concentration was determined using hexokinase in a central laboratory. Due to laboratory instrumentation changes throughout the survey time course, plasma glucose concentration was normalized using the regressions recommended by the NHANES.

Hemoglobin A1c: The high-performance liquid chromatography (HPLC) analytical column was used to determine HbA1c in a central laboratory. Since the central laboratory participated in the National Glycohemoglobin Standardization Program (NGSP) and met its criteria, the NHANES recommended that no normalization was required for HbA1c results.

Trace elements: Inductively coupled plasma dynamic reaction cell mass spectrometry was used for the determination of serum Cu, Se, and Zn concentrations in a central laboratory. All of the data was above the lower limit of detection (LLOD) for all three tests. The LLODs are as follows: serum Cu 2.5 µg/dL; serum Se 4.5 µg/L; serum Zn 2.9 µg/dL. No realignment was recommended by the NHANES for serum Cu, Se, and Zn concentrations. Based on the NHANES Laboratory Procedure Manual, the reference ranges were 20–302 μg/dL for Cu [16], 95–165 μg/L for Se [17], and 70–120 μg/dL for Zn [16].

### 2.4. Other Measures

Age was recorded in years at the time of the screening interview. Gender was based on self-reported categories from the participants as well as race/ethnicity, which were categorized as Mexican Americans, other Hispanics, non-Hispanic whites, non-Hispanic blacks, and other ethnic/racial groups. BMI (kg/m^2^) was calculated from measured weight (kg) divided by the square of standing height (meter). Active smoker was defined as using tobacco/nicotine in the last 5 days. Active alcohol consumption was defined as at least 12 alcohol drinks per year. Family history of diabetes was defined as any of the blood relatives (including father, mother, sisters, or brothers) ever informed by a health professional that they had diabetes.

Trace element intake: Trace element intake was based on two 24-h dietary recall interviews. The first dietary recall interview was collected in-person on the day of medical examination with a follow up telephone interview three to 10 days later. Only those data with dietary recall status defined as reliable and that met the minimum criteria by the NHANES were included in this study. Cu and Zn were assessed as mg per day while Se were assessed as mcg per day. -Daily total trace element intake was the sum of daily trace elements from food (daily dietary intake) and daily trace element supplements taken by participants (daily supplement).

### 2.5. Statistical Analysis

Continuous data were expressed as the mean ± standard deviation. Since serum Cu, Se, and Zn concentrations were measured in a one-third subsample of participants and as recommended by the NHANES, the data were analyzed with the consideration of special sample weights. Sample-weighted results were presented unless otherwise specified. Categorical variables were examined using the Chi-square test. Continuous variables were examined using ANOVA with the consideration of additional covariates as indicated. In Model 1, no covariate was considered. In Model 2, age, gender, and BMI were entered as covariates. In Model 3, daily trace element intake, racial/ethnic group, status of current tobacco use, status of current alcohol consumption, and family history of diabetes were entered as covariates in addition to age, gender, and BMI. Ordinary least squares regression analysis was used to assess the correlation of serum trace elements with trace element intake. The SYSTAT 13 for Windows was used for calculations in this study. A nominal *p* value of less than 0.05 was considered to be significant.

## 3. Results

### 3.1. Surveyed Subjects

Among survey participants, 15,526 subjects were identified who were aged 20 years or older with reported BMI and defined status of glucose tolerance. Since trace elements were only measured in one-third of surveyed subjects, the sample size was reduced to 5087 subjects (Table 1) based on the described sampling scheme (Figure 1). We compared the clinical characteristics between the included and excluded subjects. No difference between the two groups was found in age (*p* = 0.08), gender (*p* = 0.07), BMI (*p* = 0.60), racial/ethnic groups (*p* = 0.89), smoker status (*p* = 0.63), current alcohol consumption (*p* = 0.10), family history of diabetes (*p* = 0.13), glucose tolerance status (*p* = 0.37), established diabetes (*p* = 0.80), HbA1c (*p* = 0.24), fasting plasma glucose concentration (*p* = 0.62), and 2-h post-challenged plasma glucose concentrations (*p* = 0.92). These results suggested that the subsampling scheme for trace elements did not introduce any sampling bias. Of note, 80% of the diabetic subjects in this study were determined based on established diabetes, either by being informed to be diabetic by a healthcare provider or by using any diabetic medications.

### 3.2. Prevalence of Copper, Selenium, and Zinc Deficiency and Excess

We compared daily dietary intake, daily supplement and daily total intake of Cu, Se, and Zn by the three states of glucose tolerance (Table 2). No difference was noted among the participants with NGT, AGT, and DM, except for daily Cu supplement (*p* = 0.02).

As shown in Table 1 and Table 3, almost all participants had serum trace element concentrations within the reference range. Other than Zn deficiency, the prevalence of deficiency and excess in Cu and Se was uncommon in the study population (Table 3). Only one participant had an elevated serum Cu concentration (306.60 μg/dL) who was not taking a Cu supplement, while no study participants with serum Cu concentration below the reference range were noted.

There were 159 participants with serum Se concentrations above the reference range. The highest serum Se concentration was 297.90 μg/L. Among them, 72 participants were taking daily Se supplements ranging from 16.40 to 283.15 μg/day. There were 74 participants with serum Se concentrations below the reference range. The lowest serum Se concentration was 58.10 μg/L and, among them, 10 participants were taking daily Se supplements from 12.5 to 105.0 μg/day.

There were 68 participants with a serum Zn concentration above the reference range. The highest serum Zn concentration was 232.50 μg/dL. Among them, 23 participants were taking daily Zn supplements ranging from 3.35 to 166.23 mg/day. There were 1143 participants with serum Zn concentration below the reference range. The lowest serum Zn concentration was 31.40 μg/dL. Among them, 324 participants were taking daily Zn supplements ranging from 0.13 to 84.60 mg/day.

### 3.3. Trace Element Intake and Serum Trace Element Concentration

Data for daily dietary Cu intake was available in 78.65% of participants with an average of 0.66 mg/day. As shown in Table 4, serum Cu concentration was negatively correlated with daily dietary Cu intake (*p* < 0.0001), and with daily total Cu intake (*p* = 0.01). Thus, there was inconsistency between daily total Cu intake and serum Cu concentration, while serum Cu concentration was not a reflection of daily total Cu intake. As shown in Table 5, when compared to NGT participants, the percentage of participants taking daily Cu supplements was higher in those with AGT (*p* < 0.0001) and also with DM (*p* < 0.0001). However, the daily total Cu intake was not different among the three groups (Table 2).

Serum Se concentration was positively correlated with daily dietary Se intake (r = 0.0300, *p* = 0.03), with daily Se supplement (*p* < 0.0001), and with daily total Se intake (*p* < 0.0001). Thus, serum Se concentration was a good reflection of daily Se intake. As shown in Table 4, there were more AGT (*p* < 0.0001) and DM (*p* < 0.0001) participants who were taking Se supplementation when compared to NGT participants. However, no difference was noted in the daily total Se intake among three groups (Table 2).

Serum Zn concentration was positively correlated with daily dietary Zn intake (*p* = 0.0009), with daily Zn supplement (*p* < 0.0001), and with daily total Zn intake (*p* < 0.0001). Thus, serum Zn concentration was a good reflection of daily Zn intake. As shown in Table 4, when compared to NGT participants, there were more AGT participants (*p* < 0.0001) but less DM participants (*p* < 0.0001) taking daily Zn supplements. Again, no difference was noted in the daily total Zn intake among the three groups (Table 2).

### 3.4. Copper

The impact of glucose tolerance states on serum Cu concentration was noted after adjustment for covariates in Models 2 and 3 (a of Table 6). The primary difference was between the NGT and AGT groups (*p* = 0.01 in Model 2 and *p* = 0.03 in Model 3). This observation was coincident with the highest frequency of Cu supplement in the AGT group (Table 4). Among covariates, female gender, BMI, and non-current smoking status had significant positive impact (*p* < 0.0001), and age had a negative impact (*p* = 0.0002) on serum Cu concentration, but not with daily Cu intake.

### 3.5. Selenium

The impact of glucose tolerance states on serum Se concentration is shown in (b) of Table 6. The DM group had significantly higher serum Se concentration than both the NGT and AGT groups (*p* = 0.001 - < 0.0001). Among the covariates, daily Se intake (*p* < 0.0001) and female gender (*p* < 0.0001) had significant positive impact (*p* < 0.0001 for both), while BMI (*p* = 0.0001) and non-current smoking status (*p* = 0.0002) had a negative impact on serum Se concentrations.

### 3.6. Zinc

The impact of glucose tolerance states on serum Zn concentration was noted in all three models (c of Table 6). The highest serum Zn concentration was in the AGT group (*p* < 0.0001) followed by the DM group (*p* < 0.0001) as compared to the NGT group. Among all the covariates, daily Zn intake, and female gender (*p* < 0.0001) had significant positive impact while BMI (*p* < 0.0001) and age (*p* = 0.0003) had significant negative impact on serum Zn concentrations. The prevalence of Zn deficiency in the NGT group was 24.91% (95% CI: 24.91–24.92) which was much higher than in the AGT group (15.60%, 95% CI 15.60–15.61) and also in the DM group (18.52%, 95% CI: 18.52–18.53).

## 4. Discussion

We compared the daily intake and serum concentrations of Cu, Se, and Zn among the participants with NGT, AGT and DM in a representative U.S. population. In this population, 33.61% were overweight and 38.50% were obese among individuals aged 20 years or older. Other than Zn deficiency (21.15%), the prevalence of Zn excess and deficiency and excess in Cu and Se in the study population (Table 3) is relatively rare (<4%). The frequency of supplementary use was lowest in Se (30.12%) and highest in Zn (35.20%), while intermediate in Cu (30.64%). Daily Se and Zn intake was a significant determinant of serum Se and Zn concentrations. When compared to the NTG group, serum Cu concentration was significantly higher in the AGT group, serum Se concentration was significantly higher in the DM group, and serum Zn concentration was significantly higher in both AGT and DM groups. Thus, higher serum Cu, Se, and Zn concentrations were associated with abnormal glucose metabolism. Other than daily Cu supplant, we found no difference in daily dietary intake, daily supplementation, and daily total intake of Cu, Se, and Zn among three states of glucose tolerance, while significant differences were observed in the serum Cu, Se, and Zn concentration among three states of glucose tolerance.

In adult diabetics in this country, use of mineral supplements remained stable from 1999 to 2014 [18]. In the NHANES, the dietary data was based on dietary information provided from memory by study participants. Although widely accepted in nutritional research, this approach has weaknesses and remains controversial [19,20]. Indeed, we did observe some discrepancy in this study. For example, we noted a significant negative correlation between serum Cu concentration and intake from food and total daily intake. The discrepancy in Cu could result from the intrinsic issues with the methods used in collection of dietary information. When compared to the NGT group (29.21%), Cu supplementation was more common in the AGT (32.80%, *p* < 0.0001) and DM (31.78%, *p* < 0.0001) groups and may account for the increased serum Cu concentration in the AGT and DM groups (Table 5). Thus, the higher serum Se and Zn concentrations could be the result of daily dietary intake and/or daily supplement, but not Cu.

Cu is an essential trace element and a key constituent of the respiratory enzyme complex cytochrome c oxidase [21]. Cu is also found in superoxide dismutase [22], which reduces oxidative stress. Oxidative stress is thought to promote the development of type 2 diabetes [23]. In rats, Cu restriction resulted in impaired glucose-stimulated insulin secretion [24]. Type 2 diabetes was associated with hereditary Cu deficiency [25,26]. In patients with hereditary ceruloplasmin deficiency, pancreatic islets were found to have fewer insulin-containing beta cells [27]. However, in the present study, Cu deficiency was not associated with DM. In contrast, Cu accumulation in Wilson’s disease was associated with DM [28]. Conversely, in some individuals with Wilson’s disease, glucose intolerance with concomitant hyperactive insulin production was noted and this abated following treatment with penicillamine [29]. These data suggest that Cu plays a role in the pathogenesis of clinical diabetes. In db/db mice that spontaneously develop diabetes, serum Cu concentrations were significantly higher compared to levels in non-diabetic mice while treatment with tetra-thiomolybdate, a Cu chelating agent, reduced insulin resistance and ameliorated glucose intolerance [30]. Dietary intake of Cu was associated with a higher risk of DM in a Japanese population [31]. Given that in the present study a cohort Cu deficiency was not found and that elevated Cu was associated with ATG, Cu supplementation in the absence of deficiency is unlikely to impact the incidence of DM.

Se is also basically essential and has pleiotropic effects on reproduction, thyroid hormone metabolism, DNA synthesis, and protects from oxidative damage and infection [32]. In Se deficient db/db mice, Se supplementation improved insulin resistance [33]. In diabetic rats, Se improved glucose homeostasis and partly reversed expression of liver glycolytic and gluconeogenic enzymes [34]. In humans, elevated dietary Se intake correlated with less insulin resistance [35]. However, this was not confirmed in non-selenium deficient individuals. Following oral Se supplement (200 μg/d) for an average of 7+ years, type 2 diabetes developed more often in recipients versus controls with hazard ratio of 1.55 (95% CI: 1.03–2.33) [36]. A roughly linear trend between Se levels and the risk of DM was observed with a relative risk of 3.6 (95% CI: 1.4–9.4) for serum Se concentrations of 140 μg/L compared to Se levels <45 µg/L [9]. Furthermore, Se supplementation increased the risk of DM by 11% in mice (RR 1.11, 95% CI: 1.01–1.22) [33]. A meta-analysis revealed a non-linear dose response relationship between serum Se and the risk of DM with a positive association with serum Se concentration >125 μg/L [35]. Others found an association between Se supplementation and DM (30). Thus, high normal serum Se concentration could have a negative impact on glucose metabolism. Herein we note very few instances of Se deficiency (1.45%). Together, the findings suggest that, in attempt to reduce the incidence of DM, Se supplementation should be discouraged in adults without Se deficiency.

After iron, Zn is the second most abundant essential trace metal and is essential for the function of over 300 enzymes and 1000 transcription factors [37]. Zn is required for the processing and storage of insulin [7]. Specifically, the zinc transporter ZnT8 is vital for the biosynthesis and secretion of insulin, the uptake of zinc into insulin secretory granules, and Zn co-secretion with insulin [38]. The common ZnT8 gene polymorphism rs13266634 that involves a tryptophan-to-arginine substitution is associated with an increased risk of DM and decreased beta cell function [39]. ZnT8 is highly specific to pancreatic insulin-producing beta cells, and autoantibodies to the same are found in individuals with type 1 diabetes [40]. Zn deficiency was reported as a risk factor for diabetes [41]. However, there is a paucity of clinical studies on Zn deficiency and type 2 diabetes. Meta-analysis found no evidence to support the use of zinc supplementation for the prevention of type 2 diabetes [42]. In this study, we observed a significant association of higher serum Zn concentration in participants with abnormal glucose metabolism. In contrast, Zn deficiency (21.15%, 95% CI: 21.14–21.16) was more prevalent than Cu and Se deficiency. Of note, Zn deficiency is very common in the United States [43], as was also noted in this study. Therefore, in the present cohort, higher serum Zn concentration was associated with AGT and DM.

In the NHANES, the type of DM was not reported nor were islet antibodies determined. Since insulin treatment is relatively common in type 2 diabetes, it would not be appropriate to classify the type of diabetes based on the use of insulin alone. Thus, we were not able to separate type 1 diabetes from type 2 diabetes in this study. Based on self-reporting, the 2016 National Health Interview Survey estimated the prevalence of type 1 diabetes to be 0.55% and type 2 diabetes to be 8.6% [44], while type 2 diabetes accounted for 93.99% of all diabetes. Consistent with this, the present study included primarily individuals with type 2 diabetes. Relevant to type 1 diabetes, a role for Cu [45], Se [46], and Zn [47] in autoimmunity was suggested. In Egypt, Cu, Se, and Zn concentrations in patients with type 1 diabetes were found to be lower versus healthy controls [48]. Contrasting this, in the United States, higher serum Cu concentrations were noted in patients with type 1 diabetes than in healthy controls [49]. Nonetheless, the role of Zn and zinc transporter 8 in type 1 diabetes is well established [50]. Whether the observed relationships we found in the present study are applicable to type 1 diabetes remains to be determined.

Our study benefited from several strengths. Data was derived from a highly representative US population. As well, results were analyzed with a complicated sampling scheme as recommended by the NHANES, likely extending the findings to the general population. Furthermore, among individuals aged 20 years or older, no difference was found between BMI and defined glucose tolerance status among subjects with trace element measurement and those without trace element measurement, further assuring lack of sampling bias. Thus, our observations could be applied to the general trace element replete population.

However, our study had several limitations. Being a cross-sectional observational study, causal effect cannot be implied. The possibility that higher serum Cu and Zn concentrations reflect, rather than cause, worsening glucose homeostasis cannot be excluded. This may not be the case for Se, as the negative impact on glucose metabolism from Se supplementation was previously demonstrated [36]. Additional interventional studies, including chelation and supplementation studies, are needed to assess the role of Zn and Cu in diabetes and glucose homeostasis. Due to the nature of the NHANES, we were not able to separate type 1 diabetes out in this study. Due to its low prevalence, we felt it had no major impact on the observed results, as discussed above. The reference ranges of serum trace element concentration are highly dependent upon the nature of laboratory assays, the instruments used, and institutions/agencies. Since this study is based on the NHANES data, we quoted the reference ranges from the NHANES as described in the method sections. With different reference ranges, the prevalence of trace element deficiency and excess will definitely change. Due to the lack of reference ranges for the daily intake of Cu, Se, and Zn, the percentage of the population with such deficiencies in the dietary intake cannot be assessed. Likewise, only the serum zinc cut-off point allows us to compare this study with other similar ones, excluding Cu and Se.

Serum levels of trace elements have U-shaped dose response curves (hormesis) in biological systems [51]. This nutritional hormesis model suggests the possibility of an adverse impact on glucose homeostasis with extremes of physiological concentrations of trace elements. Further studies are required to demonstrate the causal effect and to explore the underlying molecular mechanisms.

## 5. Conclusions

To the best of our knowledge, this is the largest trace element study to date. There was a significant percentage of this population (21%) with serum zinc deficiency (hypozincemia). Daily Se and Zn intake was a significant determinant of serum Se and Zn concentrations. When compared to the NTG group, serum Cu concentration was significantly higher in the AGT group, serum Se concentration was significantly higher in the DM group, and serum Zn concentration was significantly higher in both AGT and DM groups. Although a role for Cu, Se, and Zn deficiency in the development of diabetes is well recognized, higher serum Cu, Se, and Zn concentrations appeared to be associated with abnormal glucose homeostasis. We have a very strong reservation on the recommendation of trace element supplementation in non-deficient individuals.

## Figures and Tables

**Table 1:** Clinical characteristics of study subjects.

	All Subjects			Normal Glucose Tolerance			Abnormal Glucose Tolerance			Diabetes Mellitus			*p*
N	5087			2452			1575			1060			
Age (year)	49	±	18	42	±	16	53	±	17	60	±	14	<0.0001
Gender (female)	2578		50.68%	1332		54.32%	751		47.68%	495		46.70%	<0.0001
Body mass index (kg/m^2^)	29.16	±	7.07	27.46	±	6.26	29.78	±	7.06	32.17	±	7.69	<0.0001
Current smoker	986		19.38%	477		19.45%	333		21.14%	176		16.60%	0.02
Current alcohol consumption	3691		72.56%	1876		76.51%	1099		69.78%	716		67.55%	<0.0001
Family history of diabetes	2061		40.52%	842		34.34%	566		35.94%	653		61.60%	<0.0001
Racial/ethnic group													<0.0001
Mexican Americans	692		13.60%	289		11.79%	220		13.97%	183		17.26%	
Other Hispanics	560		11.01%	240		9.79%	188		11.94%	132		12.45%	
Non-Hispanic whites	1950		38.33%	1052		42.90%	551		34.98%	347		32.74%	
Non-Hispanic blacks	1092		21.47%	468		19.09%	371		23.56%	253		23.87%	
Others	793		15.59%	403		16.44%	245		15.56%	145		13.68%	
Established diabetes	848		16.67%							848		80.00%	
Years of diabetes	12	±	11							12	±	11	
Serum copper (ug/dL)	120.1	±	30.7	118.6	±	32.7	121.0	±	28.5	122.3	±	28.9	0.002
Serum selenium (ug/L)	129.5	±	18.4	127.9	±	17.5	129.6	±	18.4	133.4	±	19.8	<0.0001
Serum zinc (ug/dL)	81.5	±	15.3	79.9	±	15.3	83.2	±	15.2	82.6	±	15.0	<0.0001

Unweighted mean ± standard deviation; n, unweighted percent.

**Table 2:** Daily dietary intake, daily supplement, and daily total intake of copper, selenium, and zinc by the states of glucose tolerance.

		Daily Dietary Intake		Daily Supplement		Daily Total Intake	
		Mean	95%CI	Mean	95%CI	Mean	95%CI
Copper (mg/day)	Normal glucose tolerance	0.68	0.66–0.70	1.24	1.12–1.36	1.06	1.01–1.10
	Abnormal glucose tolerance	0.66	0.63–0.68	1.28	1.12–1.44	1.07	1.01–1.14
	Diabetes mellitus	0.69	0.66–0.73	0.94	0.73–1.14	1.02	0.93–1.11
	*p* *	NS		0.02		NS	
Selenium (mcg/day)	Normal glucose tolerance	60.29	59.02–61.56	62.34	55.80–68.88	79.93	77.20–82.66
	Abnormal glucose tolerance	61.64	59.85–63.42	66.46	57.26–75.65	82.64	78.80–86.48
	Diabetes mellitus	60.35	57.97–62.74	62.00	50.34–73.66	82.03	76.91–87.16
	*p* *	NS		NS		NS	
Zinc (mg/day)	Normal glucose tolerance	6.01	5.86–6.17	13.57	12.61–14.53	11.00	10.52–11.48
	Abnormal glucose tolerance	5.96	5.74–6.18	14.82	13.47–16.17	11.44	10.76–12.12
	Diabetes mellitus	5.96	5.67–6.25	14.31	12.51–16.11	11.25	10.34–12.15
	*p* *	NS		NS		NS	

Sample weighted mean with 95% confidence intervals; * Weighted *p* values by ANOVA; NS, not significant.

**Table 3:** Prevalence of excess and deficiency in copper, selenium, and zinc.

		Unweighted		Weighted	
	Total	n	%	%	95% CI
Cu < 20 μg/dL	5087	0	-	-	-
Cu > 302 μg/dL	5087	1	0.02%	0.02%	0.02–0.02 -
Se < 95 μg/L	5087	74	1.45%	1.46%	1.46–1.46 -
Se > 165 μg/L	5087	159	3.13%	3.02%	3.02–3.02
Zn < 70 μg/dL	5087	1143	22.47%	21.15%	21.15–21.16
Zn > 120 μg/dL	5087	68	1.34%	1.50%	1.50–1.50

**Table 4:** Correlation of serum copper, selenium, and zinc concentrations with daily dietary intake, daily supplement, and daily total intake.

	Serum Cu Concentration		Serum Se Concentration		Serum Zn Concentration	
	r	*p*	r	*p*	r	*p*
Daily dietary intake	−0.0904	<0.0001	0.0300	0.03	0.0466	0.0009
Daily supplement	0.0055	NS	0.1337	<0.0001	0.1353	<0.0001
Daily total intake	−0.0349	0.01	0.1216	<0.0001	0.1426	<0.0001

NS, not significant.

**Table 5:** Daily supplement of copper, selenium, and zinc by the states of glucose tolerance.

Trace Element	Glucose Tolerance Status	Unweighted			Weighted		
		Total	Taking Supplement		Taking Supplement		*p*
		N	n	%	%	95% CI	
Copper	Normal glucose tolerance	2452	636	25.94%	29.21%	29.20–29.22	<0.0001
	Abnormal glucose tolerance	1575	443	28.13%	32.80%	32.79–32.81	
	Diabetes mellitus	1060	276	26.04%	31.54%	31.52–31.55	
Selenium	Normal glucose tolerance	2452	619	25.24%	29.01%	29.00–29.02	<0.0001
	Abnormal glucose tolerance	1575	430	27.30%	31.78%	31.77–31.79	
	Diabetes mellitus	1060	275	25.94%	30.88%	30.87–30.90	
Zinc	Normal glucose tolerance	2452	778	31.73%	34.60%	34.59–34.60	<0.0001
	Abnormal glucose tolerance	1575	495	31.43%	37.08%	37.07–37.09	
	Diabetes mellitus	1060	302	28.49%	33.90%	33.89–33.92	

**Table 6:** Comparison of serum copper, selenium, and zinc concentrations by the states of glucose tolerance.

		Normal Glucose Tolerance	Abnormal Glucose Tolerance	Diabetes Mellitus	*p*
	n	2452	1575	1060	
(a) Copper (μg/dL)					
Model 1	Weighted mean	117.83	119.63	119.68	NS
	95% CI	(116.69–118.96)	(118.08–121.19)	(117.63–121.74)	
Model 2	Weighted mean	117.71	120.28	118.93	0.02
	95% CI	(116.67–118.76)	(118.89–121.66)	(117.01–120.85)	
Model 3	Weighted mean	117.86	120.07	118.81	0.05
	95% CI	(116.81–118.91)	(118.68–121.45)	(116.87–120.75)	
(b) Selenium (μg/L)					
Model 1	Weighted mean	128.91	130.32	132.92	<0.0001
	95% CI	(128.22–129.60)	(129.38–131.27)	(131.67–134.17)	
Model 2	Weighted mean	129.16	130.02	132.62	<0.0001
	95% CI	(128.44–129.88)	(129.07–130.97)	(131.30–133.93)	
Model 3	Weighted mean	129.01	130.17	132.85	<0.0001
	95% CI	(128.29–129.72)	(129.23–131.12)	(131.53–134.18)	
(c) Zinc (μg/dL)					
Model 1	Weighted mean	80.22	84.94	82.84	<0.0001
	95% CI	(79.66–80.79)	(84.16–85.71)	(81.82–83.87)	
Model 2	Weighted mean	79.84	85.14	83.76	<0.0001
	95% CI	(79.25–80.43)	(84.36–85.91)	(82.68–84.84)	
Model 3	Weighted mean	79.82	85.08	83.93	<0.0001
	95% CI	(79.23–80.40)	(84.30–85.85)	(82.85–85.01)	

Model 1, unadjusted; Model 2, adjusted for age, gender, and BMI; Model 3, adjusted for age, gender, BMI, daily trace element intake, racial/ethnic group, current smoker, current alcohol consumption, and family history of diabetes.

## Data Availability

All data analyzed in this study are publicly available (https://wwwn.cdc.gov/nchs/nhanes/default.aspx) accessed on 24 October 2019.
